# Physics-informed TVAE workflow for data augmentation, mechanical validation, optimization of CFRP-strengthened CFST beams

**DOI:** 10.1016/j.mex.2026.103812

**Published:** 2026-02-02

**Authors:** Muluken Bogale Admasu, Addisu Mengistu Admassu, Tariku Habtamu Biresaw, Abrham Gebre Tarekegn

**Affiliations:** a Faculty of Civil Engineering, Arba Minch Institute of Technology, Arba Minch University, P.O. Box 21, Arba Minch, Ethiopia; b Computer Science, College of Natural and Computational Sciences, Addis Ababa University, Addis Ababa, Ethiopia; c School of Civil and Environmental Engineering, College of Technology and Built Environment, Addis Ababa University, Addis Ababa, Ethiopia

**Keywords:** Concrete-filled steel tube, Carbon fiber reinforced polymer, Physics-informed tabular Variational Autoencoder, Fiber element analysis, Multi-objective Optimization

## Abstract

This article presents a reproducible, physics-informed workflow designed to address data scarcity in the analysis and design of carbon fiber-reinforced polymer (CFRP)-strengthened concrete-filled steel tube (CFST) members. Conventional tabular generative models can reproduce statistical trends but cannot ensure that generated samples satisfy the physical, geometric, and material constraints required for structural engineering applications. To overcome this limitation, the proposed method embeds physics-based constraints directly into the data augmentation process and incorporates mechanics-based validation using nonlinear moment-curvature analysis to verify the physical admissibility of synthetic samples. The resulting datasets are suitable for downstream predictive modeling, design optimization, and decision support in data-scarce structural applications. The workflow further integrates ensemble machine learning models, explainable analysis, multi-objective optimization, and uncertainty quantification into a unified methodological framework. To support reproducibility and practical adoption, the complete methodology is implemented and released as an open-source, Streamlit-based software tool.

• Generates synthetic structural datasets that preserve statistical fidelity while enforcing fundamental physical, geometric, and material constraints for CFRP-strengthened CFST members.
• Enables design-oriented prediction, interpretation, and multi-objective optimization of CFRP configurations through an integrated ensemble machine learning framework.
• Provides an open-source, deployable software implementation to support reproducibility and practical engineering decision-making for flexural design exploration of CFRP-strengthened CFST beams.

## Graphical abstract

**Figure:**
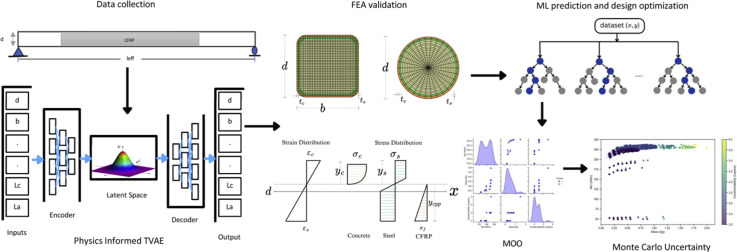

**Specifications table**

**Table:** 

Subject area	Engineering
More specific subject area	Design Optimization in Structural Engineering
Name of your method	Physics-informed Tabular Variational Autoencoder-based workflow for design optimization for CFRP-strengthened CFST members
Name and reference of original method	D. P. Kingma and M. Welling, “Auto-encoding variational bayes,” 2nd Int. Conf. Learn. Represent. ICLR 2014 - Conf. Track Proc., no. M1, pp. 1–15, 2014.
Resource availability	Code repository: [https://github.com/Crackingastro/CFRP-CFST-Flexure]

## Background

A persistent methodological difficulty in structural engineering is the limited availability of high-quality experimental data for complex composite systems, including CFRP-strengthened concrete-filled steel tube (CFST) members [[1], [2], [3], [4]]. Experimental programs for such systems require specialized facilities, extensive material characterization, and repeated loading protocols, making them costly and time-intensive. Consequently, most published datasets remain relatively small, which limits their direct applicability in data-driven modeling approaches, particularly machine learning (ML), whose predictive performance depends strongly on data volume, diversity, and coverage of the design space [3,[5], [6], [7]]. In response to this limitation, recent studies have explored a range of experimental, numerical, and data-driven strategies to enhance the understanding and prediction of CFRP-strengthened CFST behavior.

Recent investigations on CFRP-strengthened CFST members have advanced the understanding of structural behavior and performance through experimental testing, numerical simulation, and data-driven modeling. Lu et al [8] experimentally examined the flexural behavior of CFRP-strengthened CFST beam connections, while Sabih et al [9] employed finite element analysis to study the axial performance of CFRP-strengthened CFST columns. Tran et al [10] applied a CTGAN-based generative model to synthesize data for predicting ultimate axial strength and assessed reliability via Monte Carlo simulation. Hybrid frameworks combining experimental results, finite element simulations, and data-driven models have also been proposed to predict flexural capacity, such as a finite element-artificial neural network approach for circular CFRP-strengthened CFST members by Ou et al [11], as well as data-augmented modeling of CFRP-CFST behavior under concentric and eccentric loading by Xu et al [12]. Despite these advances, most recent contributions remain constrained by limited experimental datasets and focus primarily on prediction or analysis, without explicitly addressing physics-informed data augmentation, mechanics-based validation of synthetic data, or integrated multi-objective optimization under constructability considerations. These gaps motivate the development of a reproducible, physics-informed workflow capable of expanding data accessibility while preserving mechanical admissibility and design relevance.

Data augmentation using generative models, such as Tabular Variational Autoencoders (TVAEs) [13,14], has emerged as a practical strategy to mitigate data scarcity. However, conventional TVAEs learn only the statistical structure of the observed data and do not explicitly encode physical laws, geometric compatibility, or material behavior. In structural engineering applications, this limitation is critical, as statistically plausible synthetic samples may still violate fundamental mechanical principles or practical design constraints, thereby undermining their reliability for analysis or design tasks [15].

To ensure physical admissibility of augmented data, domain knowledge must be integrated directly into the data-generation process rather than applied only as post-processing. This requires embedding engineering constraints during model training and enforcing them during sampling, followed by mechanics-based validation of the generated samples. For the behavior of composite members, nonlinear moment-curvature analysis provides a physically grounded validation mechanism, allowing for the direct assessment of stiffness evolution, strength development, and consistency of post-peak response in synthetic beam configurations [16].

Beyond data generation, practical design-oriented applications require a structured and reproducible workflow that integrates predictive modeling, interpretability, and optimization. This includes training ensemble ML models on physically admissible augmented datasets, interpreting model predictions using explainable ML techniques, and performing multi-objective optimization to explore trade-offs between structural capacity, material usage, and constructability. Incorporating uncertainty quantification further enables assessment of the robustness and stability of optimized solutions under realistic variability in inputs and model responses [16].

This article presents a modular and reproducible methodology that addresses these requirements through physics-informed data augmentation, multi-level validation, predictive modeling, and optimization. Although the implementation focuses on CFRP-strengthened CFST beams, the proposed framework is general in nature and can be readily adapted to other structural systems by redefining the governing constraints, constitutive models, and validation procedures.

## Method details

To address the limited availability of high-quality experimental data for CFRP-strengthened CFST beams and to enable reliable predictive and optimization workflows, the proposed method integrates three components:

• a physics-informed generative model that augments the dataset while enforcing structural constraints,
• an explainable ensemble machine-learning predictor used as a surrogate for the flexural capacity, and
• a multi-objective optimization framework supported by uncertainty quantification.

**Fig. 1:**
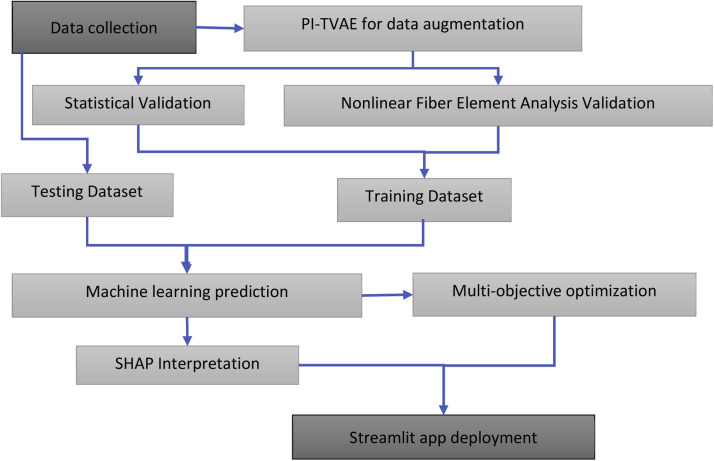
Methodological workflow for flexural design of CFRP-strengthened CFST members.

Together, these components form a reproducible computational pipeline for generating physically admissible synthetic samples, predicting flexural strength across a wide design space, and identifying efficient CFRP configurations. The following sections describe each methodological component in detail, beginning with the Physics-Informed Tabular Variational Autoencoder (PI-TVAE). Fig. 1 illustrates the overall methodological workflow adopted in this study.

### PI-TVAE for data augmentation

Standard Tabular Variational Autoencoders (TVAEs) effectively learn joint statistical distributions and generate synthetic tabular data that follow observed trends. However, for structural engineering applications, purely statistical generation is insufficient because it does not explicitly enforce physical feasibility. In datasets involving CFRP-strengthened CFST members, variables such as geometric ratios, material strengths, and stability-related limits must satisfy basic mechanical and design constraints. Without these restrictions, statistically plausible samples may still represent configurations that are structurally unrealistic or inadmissible.

To address this limitation, a physics-informed TVAE (PI-TVAE) was developed by extending the standard TVAE implementation in the Synthetic Data Vault (SDV) framework. The key modification is the explicit incorporation of engineering constraints during both the model training and post-sampling, ensuring that generated data preserve statistical fidelity while remaining physically admissible for structural analysis.

#### Architecture and preprocessing

The PI-TVAE retains the encoder-latent-decoder architecture of the SDV TVAE framework. Input data consists of mixed numerical and categorical variables describing CFRP-strengthened CFST beam properties. Numerical and categorical features are identified using the SingleTableMetadata utility. Categorical variables related to CFRP strengthening configuration are explicitly defined before training to preserve discrete engineering design variables and prevent distortion during encoding.

#### Physics-Informed latent space filtering

Before decoding, latent samples are screened using a binary physics indicator to remove configurations violating essential structural limits. The enforced constraints are:

• 12 ≤ d/ts ≤ 50 (local stability control) [[17], [18], [19]].
• 235 ≤ fsyd ≤ 700 (Steel strength range) [20].
• 12 ≤ fcu ≤ 120 (concrete compressive strength range) [20].
• leff/d ≤ 4 (member slenderness control) [21].

The constraint bounds applied during latent space filtering were selected to define a feasible design domain consistent with established CFST practice, provisions of international design codes, and reported experimental evidence. The diameter-to-thickness ratio (d/ts) is limited to control local instability of thin steel tube walls under flexural loading, in accordance with experimentally observed stability limits and design code recommendations [[17], [18], [19]]. The steel yield strength (fsyd) interval encompasses commonly used structural steel grades in CFST members, aligned with strength classes specified in widely adopted international design standards (e.g., Eurocode 4) and representative studies [20]. Similarly, the concrete compressive strength (fcu) bounds reflect the normal- to high-strength concretes typically adopted in CFST systems, ensuring realistic stiffness and confinement behavior while avoiding strength levels requiring alternative constitutive assumptions [20]. The effective slenderness limit (leff/d) is introduced to suppress global instability effects, consistent with conservative stability provisions in composite member design standards [21].

Collectively, these constraints establish a physically admissible latent design space, whereas CFRP strengthening parameters are treated as controllable design variables rather than feasibility constraints, reflecting their role as externally applied retrofit measures.

This latent-level filtering mirrors the “latent variable → physics indicator” block in the proposed workflow and ensures that physically inconsistent representations are excluded before data reconstruction.

#### Physics-Penalized decoder loss

During training, the standard TVAE loss is augmented with a physics-based penalty to discourage violation of engineering constraints. The total loss is defined as:

(1) $$L_{total}=L_{base}+\lambda_{2}\left(t\right)\;\times L_{phy}$$

Where L_base_ is the original TVAE loss (reconstruction error and Kullback-Leibler divergence), L_phy_ is a normalized penalty aggregating constraint violations, and λ_2_(t) is a time-dependent weight.

**Fig. 2:**
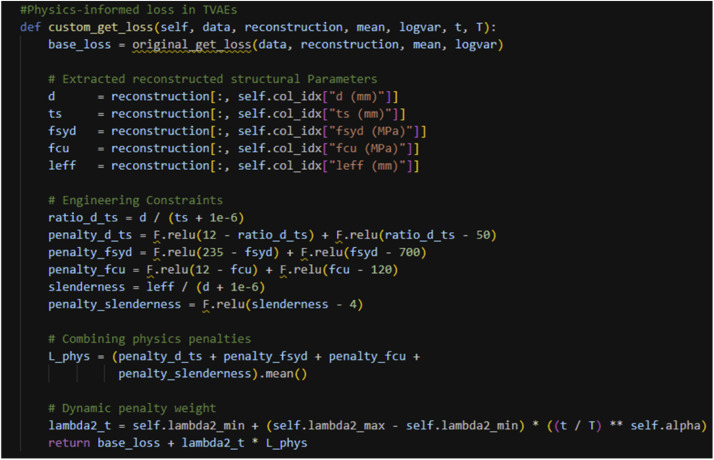
Custom physics-informed loss inside the modified TVAE class.

The penalty term is implemented using rectified linear unit (ReLU) functions to penalize values outside admissible ranges. The weighting factor λ_2_(t) follows an annealing schedule that increases progressively during training, allowing the model to first learn the statistical structure of the data and subsequently enforce physical consistency. Fig. 2 shows the implementation of the physics-based penalty within the modified TVAE decoder loss.

#### Physics-Informed sampling

**Fig. 3:**
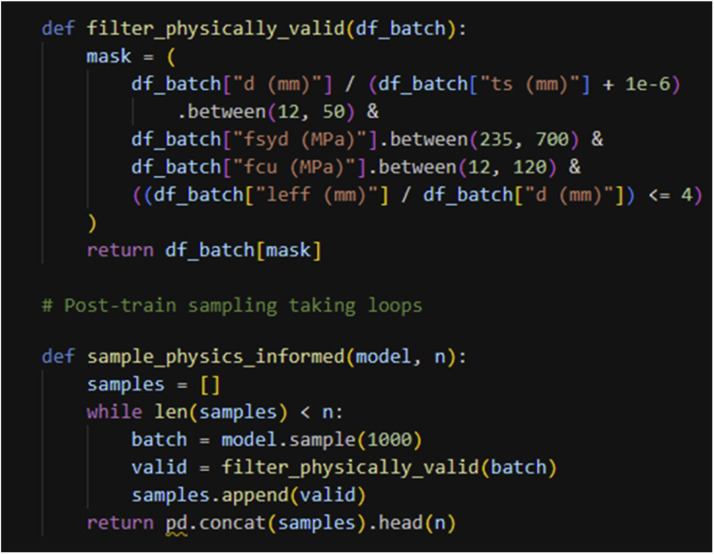
Implementation of physics-informed sampling with iterative validity checking against structural design constraints.

After training, a rejection-based sampling procedure guarantees that all generated synthetic samples satisfy the prescribed mechanical, geometric, and stability constraints. The trained PI-TVAE first produces raw samples, which are then iteratively filtered using the same constraint set applied during training. Only samples meeting all physical requirements are retained. This two-stage enforcement-soft constraint penalization during training and hard constraint filtering during sampling ensures physical admissibility of the final synthetic dataset. Fig. 3 presents the code-level implementation of the physics-informed sampling procedure applied after training.

#### Hyperparameter optimization

Model hyperparameters, including embedding dimension, network width, batch size, number of training epochs, and maximum penalty weight, are optimized using the Optuna framework. Each trial trains a PI-TVAE, generates physics-filtered synthetic samples, and evaluates similarity to the original dataset using the mean Kolmogorov-Smirnov (KS) statistic across all variables. The configuration minimizing the average KS statistic was selected for final model training; the resulting parameter values are reported in the companion article [16].

#### Statistical checks embedded in the PI-TVAE workflow

As part of the PI-TVAE workflow, statistical consistency checks are embedded to verify the preservation of distribution and dependency. A full validation of these checks is reported in the method validation section.

#### Reproducibility notes

The method is implemented in Python using PyTorch, the SDV library, and Optuna. Reproducibility is ensured by fixing a random seed for Python, NumPy, and PyTorch. All components of the workflow are fully executable on standard CPU hardware, ensuring accessibility and reproducibility. In the present study, GPU acceleration was employed to reduce training and optimization time; however, this does not affect the methodological steps or numerical outcomes.

### Nonlinear fiber element analysis validation

This component validates the physics-informed synthetic dataset by evaluating whether each synthetic parameter set produces a realistic flexural response under nonlinear bending. A fiber-section numerical procedure is implemented for circular, square, and rectangular CFRP-CFST beams, enabling the generation of ensembles of moment-curvature (M-ϕ) curves from synthetic samples. These curves are then aggregated into uncertainty bands to quantify variability in flexural response. The analysis:

• Uses experimentally calibrated constitutive laws
• Builds a fiber-discretized section
• Iteratively enforces force equilibrium at each curvature increment
• Produces an M-ϕ curve for each synthetic parameter set.
• Aggregates all curves to compute median and 5-95% uncertainty bands, validating the global and post-peak consistency of synthetic data.
• Compares the ensemble response against the experimental M-ϕ curve for the target specimen.

This step provides mechanics-based validation, complementing the statistical test performed earlier. Quantitative comparisons and interpretation of these uncertainty bands are reported in the method validation section.

#### Input data and parameter extraction

Synthetic parameter sets are read from an Excel sheet (SyntheticParams). Each row corresponds to one beam. Missing values default to experimentally measured specimen parameters. A corresponding experimental M-ϕ curve is read from a separate sheet (Experimental) for later comparison.

Each row triggers a parameter-setup function—setup_section_from_row ()— which computes geometric variables, confinement pressure, ultimate concrete strength, and ultimate strain following standard confinement models.

#### Material constitutive models

Three nonlinear constitutive laws are used consistently across circular and rectangular sections:

• Concrete (confined core): A piecewise nonlinear compressive model capturing pre-peak parabolic behavior and post-peak softening [22].
• Steel tube: Elastic-plastic with strain hardening and optional fracture parameters.
• CFRP: Linear elastic up to rupture, followed by a brittle drop or softened tail.

These models ensure realistic load distribution among fibers during progressive bending.

#### Fiber discretization

Circular CFRP-CFST Section

• Radial-angular mesh: n_radial_ * n_ϴ_ = 180 * 180 fibers
• Region assignment: Inner → concrete; middle ring → steel; outer ring → CFRP
• Fiber area: A = r * ∆r * ∆ϴ

Where n_radial_ and n_ϴ_ denote the number of divisions in the radial and circumferential directions, respectively, and ∆r and ∆ϴ are the radial and angular discretization steps.

Rectangular CFRP-CFST Section

• Uniform 2D grid of n_x_ * n_y_ = 400 * 400 fibers
• Region assignment via geometric checks (steel shell, concrete core, CFRP laminate)
• Fiber area: A = ∆x * ∆y

Where n_x_ and n_y_ denote the number of subdivisions along the width and depth directions, and ∆x and ∆y represent the grid spacing in each direction.

This discretization offers a balance between accuracy and computational efficiency for ensemble-level analysis.

#### Moment-Curvature solution algorithm

For each synthetic beam section, curvature is prescribed indirectly by incrementing the extreme fiber strain ε_fb_. The neutral axis depth, x_c_, is solved iteratively using a bisection method to satisfy axial equilibrium:

(2) $$C\left(\varepsilon_{fb},\;x_{c}\right)=T\left(\varepsilon_{fb},\;x_{c}\right)$$

Where C and T denote the resultant compressive and tensile forces obtained by integrating fiber stress across the section.

At convergence, the Section curvature ϕ is evaluated as:

(3) $$\begin{array}{c} \phi =\frac{\varepsilon_{\mathrm{fb}}}{h-x_{c}}\left(\mathrm{rect}\mathrm{angu}\mathrm{lar}\;\mathrm{sect}\mathrm{ion}\right),\;\phi =\frac{\varepsilon_{\mathrm{fb}}}{D-x_{c}}\;\left(\mathrm{circ}\mathrm{ular}\;\mathrm{sect}\mathrm{ion}\right) \end{array}$$

Where h and D represent the total section depth and diameter, respectively.

The corresponding bending moment is computed using fiber integration:

(4) $$M=\sum_{i}\sigma_{i}A_{i}y_{i}$$

Where σ_i_, A_i_, and y_i_ denote the stress, area, and distance from the section centroid of the i-th fiber.

The procedure continues until the CFRP rupture strain is exceeded or the concrete reaches its ultimate strain. Each synthetic row yields one full M-ϕ curve.

#### Ensemble-Based uncertainty-band computation

All synthetic curves are interpolated onto a common curvature grid (300 points). For each curvature level, the following statistics are computed:

• Median M50
• Lower percentile M5
• Upper percentile M95

These produce a shaded uncertainty band, representing model dispersion and physical variability captured by PI-TVAE. A representative synthetic curve is selected as the one whose ultimate moment is closest to the ensemble mean.

### Machine learning prediction using optimized Ensemble models

An ensemble machine-learning workflow was implemented in Python to predict the flexural strengths of CFRP-strengthened CFST beams. The workflow includes Random Forest, AdaBoost, Gradient Boosting, XGBoost, and a Stacking regressor, selected to represent commonly used tree-based and boosting-based ensemble methods for nonlinear regression problems in structural engineering.

Hyperparameter optimization for all models was conducted using the Optuna framework. The optimized parameters include the number of estimators, learning rate, maximum tree depth, feature sampling strategy, boosting-related parameters, and regularization terms specific to XGBoost. A repeated 10-fold cross-validation scheme was applied during the optimization process.

Model training was performed exclusively using synthetic data generated by the PI-TVAE model, while experimentally obtained data were reserved for independent evaluation. This data separation strategy was applied consistently across all ensemble models to avoid information leakage and reflect data-scarce engineering scenarios.

Model evaluation procedures employed standard regression metrics, including the coefficient of determination (R^2^), mean absolute error (MAE), mean absolute percentage error (MAPE), and root mean square error (RMSE). Residual analysis was conducted to assess prediction consistency and to detect potential systematic bias across the range of flexural strengths. For model explainability, SHAP (Shapley Additive explanations) analysis was applied to the optimized ensemble models to quantify the contribution of each input parameter to the predicted flexural strength, supporting transparency and informed downstream optimization.

### Multi-objective optimization framework

#### Objective and optimization setup

A three-objective optimization framework was implemented to explore CFRP strengthening configuration for CFRP-strengthened CFST beams [23]. The objectives were defined as follows:

• Maximize flexural capacity (Mu) predicted by the trained ensemble surrogate model.
• Minimize CFRP mass (mass), computed from geometric and material inputs.
• Minimize constructability demand (constructability), represented by the total number of CFRP layers (Longitudinal (Lc)+ Transverse (Tc))

Optimization was performed using the Optuna framework with a Tree-structured Parzen Estimator (TPE) sampler (random seed = 42). For each trial, a candidate CFRP configuration was evaluated by the surrogate model, and the objective triplet (Mu, mass, constructability) was returned. Trials were executed with user-defined parallelism (n_jobs = 1 or -1).

#### Design variables and fixed parameters

The optimization design variables were defined as:

• Lc ϵ [1,4],
• La ϵ {0.25, 0.5, 0.75, 1.0}
• Tc ϵ [1,2],
• Ta ϵ {0.25, 0.5, 0.75, 1.0}

Where La and Ta represent the wrapped-area ratios for longitudinal and transverse CFRP, respectively.

Fixed parameters include section geometry, steel and concrete material properties, effective length (leff), and CFRP material parameters supplied through the Streamlit interface.

A standard multi-objective Optuna study with objective directions (maximize, minimize, minimize) was used to generate the Pareto-optimal solution set.

#### CFRP mass and constructability computation

For each candidate configuration, CFRP mass is computed directly from the wrapping geometry:

Circular section:

(5) $$mass=\pi *d*leff*tc\left(Lc*La+Tc*Ta\right)*\rho_{CFRP}$$

Rectangular section:

(6) $$mass=2\left(b+d\right)*leff*tc\left(Lc*La+Tc*Ta\right)*\rho_{CFRP}$$

Where tc is CFRP thickness and ρ_CFRP_ = 1600 kg/m^3^.

Constructability was quantified as: constructability = Lc + Tc

Providing a simplified and consistent representation of installation effort across all optimization runs.

#### Pareto set extraction and compromise solution

Upon completion of all trials, Pareto-optimal solutions were extracted from the study.best_trials. The following post-processing steps were applied:

• Construction of a DataFrame containing Mu, mass, constructability, and design variables.
• Min-max normalization of each objective.
• Computation of Euclidean distance to the ideal point: (Mu = 1, mass = 0, constructability = 0)
• Identification of the compromise solution as the configuration with the minimum distance.

This compromise solution was highlighted in the visualization output.

#### Visualization methods

Pareto solutions were visualized using a pairplot (seaborn) showing Mu (kNm), CFRP mass (kg), and Constructability (layers). Pareto points were marked in blue, and the compromised solution was marked in red. Figures were exported as high-resolution PNG files to ensure reproducibility.

#### Monte Carlo-based robustness assessment

A Monte Carlo extension was implemented to evaluate the robustness of the Pareto front under input uncertainty [24,25]. The procedure involved:

• Selection of the five most influential parameters identified through SHAP analysis.
• Application of stochastic perturbations:
  • fsyd ± 10%
  • d ± 5%
  • b ± 5%
  • La ± 10%
  • leff ± 2%
• Execution of a new multi-objective optimization (200 trials) for each Monte Carlo realization.
• Aggregation of Pareto-optimal solutions across all runs.
• Visualization of robustness using a kernel density estimate (KDE) plot of Mu vs CFRP mass, color-coded by constructability.

This procedure provides an uncertainty-aware representation of the optimization landscape and supports assessment of solution stability under realistic parameter variability.

#### Streamlit deployment

An interactive Streamlit-based application was developed to operationalize the proposed workflow for CFRP-strengthened CFST beams. The application provides three main functionalities:

• Flexural strength prediction,
• CFRP configuration through multi-objective optimization, and
• Monte Carlo-based robustness evaluation using a trained ensemble ML model.

#### Software and computation resources

The application was implemented in Python (version ≥ 3.8). The main dependencies include:

• Core libraries: numpy, pandas,
• Machine learning: scikit-learn, xgboost
• Optimization: optuna
• Visualization: matplotlib, seaborn
• Deployment: streamlit
• Model persistence: joblib.

All required files are placed within a single directory, including:

• app.py: Streamlit application script
• Trained ensemble models stored as serialized files (*.pkl)

The application is launched using the command: streamlit run app.py

#### Method summary and application workflow

The Streamlit application integrates all methodological components developed in this study, enabling reproducible execution through a graphical user interface.

Ensemble model loading

The user selects a pre-trained ensemble model (*.pkl). Predictions are performed using the same feature structure, preprocessing steps, and input definitions described in the ML methodology section, ensuring consistency between model training and deployment.

Single Prediction Module

The user inputs beam geometry, material properties, and CFRP strengthening parameters. The application performs the following steps:

• Validates CFRP configuration inputs against admissible ranges,
• Formats inputs into a single-row pandas DataFrame,
• Predicts flexural capacity using the selected trained ensemble model,
• Applies the β-calibration factor corresponding to the selected design code.

The predicted flexural capacity is returned in units of kNm.

Multi-Objective Optimization Module

A multi-objective optimization routine is executed using Optuna within the application. The optimization problem is defined consistently with the framework described earlier, with the objective to:

• Maximum flexural strength,
• Minimize CFRP mass,
• Minimize Constructability (layer count).

The optimization variables include longitudinal and transverse CFRP parameters (Lc, La, Tc, Ta). The application returns:

• The Pareto-optimal solution set,
• The Euclidean-distance-based compromise solution,
• Pairplot visualizations of the Pareto plots.

Monte Carlo robustness evaluation

A Monte Carlo-based robustness check is implemented to assess the stability of optimized solutions under input uncertainty. The procedure:

• Applies stochastic perturbations to selected key input parameters within predefined tolerance ranges,
• Executes a reduced MOO for each Monte Carlo realization,
• Aggregates the resulting Pareto solutions across all realizations.

The robustness of solutions is visualized using scatter plots and KDE plots in the objective space.

### Computational costs and scalability

All stages of the proposed workflow were executed on a standard GPU-accelerated workstation. The following order-of-magnitude runtime estimates are provided to assist practitioners in assessing scalability.

PI-TVAE Training: The computational cost is primarily governed by the latent dimension, batch size, number of epochs, and penalty weights. Typical training times range from tens of minutes to a few hours.

Nonlinear Fiber Element-Based Validation: This step is applied to each synthetic batch generated and depends critically on the material nonlinearity and the chosen fiber discretization. Runtime per batch varies from less than one minute for coarse resolutions to several minutes for finer discretization, with the most detailed cases requiring on the order of one hour per batch.

Multi-Objective Optimization: For a typical optimization run comprising approximately 500 trials, the process completes within less than one to a few minutes. Associated Monte Carlo-based robustness checks are similarly lightweight, requiring on the order of one to five minutes.

Note: Generation of uncertainty-band moment-curvature plots for synthetic datasets is an auxiliary post-processing step, typically requiring on the order of one hour for a medium fiber resolution.

Overall, the fiber element-based validation of synthetic batches represents the most computationally intensive stage when high-resolution analysis (e.g., ≥ 400 × 400 fibers for rectangular sections or equivalent polar discretization) is required. However, the workflow exhibits favorable practical scalability, as this validation is naturally parallelizable across independent batches, and all other components are relatively efficient.

## Method validation

The proposed workflow, comprising physics-informed data augmentation, ensemble learning, and multi-objective optimization, was validated to demonstrate methodological soundness, physical admissibility, and robustness of the implemented procedures. Validation was conducted in three sequential stages corresponding to the core components of the framework. Detailed numerical outcomes and comparative performance are reported separately in the associated research article [16].

### Stage 1: Statistical fidelity of the augmented dataset

The statistical validity of the PI-TVAE-generated synthetic data was assessed to verify preservation of the joint and marginal characteristics of the original dataset.

Procedure:

Correlation matrices and empirical cumulative distribution functions (ECDFs) were computed for both the original and synthetic datasets across all variables.

Validation metrics:

• Element-wise differences between correlation matrices were examined to assess preservation of multivariate dependencies.
• Distributional similarity was quantified using the two-sample Kolmogorov-Smirnov (KS) statistic for each variable.

These checks ensure that the physics-informed generative process maintains statistical consistency while enforcing embedded engineering constraints.

### Stage 2: Mechanical plausibility via nonlinear fiber element analysis

To verify that the synthetic parameter sets correspond to physically admissible structural configurations, a mechanics-based validation was performed.

Procedure:

Each synthetic beam configuration was propagated through a nonlinear fiber-section moment-curvature analysis. The analysis employs calibrated constitutive models for confined concrete, steel tubes, and CFRP laminates, while enforcing axial force equilibrium at each curvature increment.

Validation metrics:

The ensemble of synthetic M-ϕ responses was aggregated to compute percentile-based response envelopes (5th, 50th, and 95th percentiles). Mechanical plausibility was assessed by verifying that experimentally obtained M-ϕ responses from representative beams fall within these envelopes.

This step confirms that the generated synthetic data lead to mechanically consistent stiffness evolution, strength development, and post-peak behavior.

### Stage 3: Robustness of the predictive and optimization workflow

Surrogate model validation

The ensemble-based surrogate models used within the optimization framework were validated to ensure predictive stability, absence of systematic bias, and interpretability.

Procedure:

Ensemble modes trained on PI-TVAE-generated synthetic data were evaluated using held-out experimental beam data. Model performance was assessed using standard regression indicators (R^2^, MAE, RMSE, and MAPE).

Residual diagnostics:

Residual errors were analyzed to verify that prediction errors were randomly distributed and did not exhibit systematic trends with respect to flexural capacity. This step ensures that the surrogate models satisfy the assumptions required for reliable downstream optimization.

Explainability assessment:

Shapley Additive explanations (SHAP) were computed to quantify the contribution of each input variable to model predictions. Global SHAP importance rankings were used to confirm that dominant predictors align with established structural mechanics principles, thereby validating the physical interpretability of the learned relationships.

Multi-Objective optimization validation

The optimization module was validated to confirm stable behavior and meaningful trade-offs.

Pareto-front coherence:

The resulting Pareto-optimal solutions were inspected to verify expected engineering trade-offs between flexural strength, CFRP mass, and constructability.

Robustness under uncertainty:

Monte Carlo perturbations were applied to SHAP-identified influential parameters within realistic tolerance bounds. The resulting distribution of Pareto fronts was examined to assess clustering and stability of optimal solutions under input uncertainty.

This procedure confirms that the optimization workflow remains robust and interpretable when subjected to realistic variability in design parameters.

## Limitations

The method’s structural fidelity depends directly on the accuracy and completeness of the expert-defined physical constraints used in the PI-TVAE. Constructability is represented through a simplified layer-count metric, defined as the sum of longitudinal and transverse CFRP layers. While this metric provides a tractable and consistent proxy for implementation effort within the multi-objective optimization framework, it does not explicitly account for additional practical factors such as labor time, anchorage detailing, surface preparation, or installation sequencing. Incorporation of such factors would require project-specific data and more detailed construction modeling, which is beyond the scope of the present study. Future extensions could therefore explore composite constructability indices that integrate layer count with these additional considerations. The uncertainty analysis is similarly limited to bounded perturbations of selected input variables. Together, these modeling choices define the current scope of applicability while highlighting opportunities for future refinement.

## Ethics statements

Our work did not involve human subjects, animal experiments, or data collected from social media platforms.

## CRediT authorship contribution statement

**Muluken Bogale Admasu:** Conceptualization, Methodology, Software, Validation, Data curation, Visualization, Writing – original draft, Writing – review & editing. **Addisu Mengistu Admassu:** Methodology, Formal analysis, Validation, Writing – review & editing. **Tariku Habtamu Biresaw:** Visualization, Writing – review & editing. **Abrham Gebre Tarekegn:** Methodology, Writing – review & editing.

## Declaration of competing interest

The authors declare that they have no known competing financial interests or personal relationships that could have appeared to influence the work reported in this paper.

## Data Availability

Data will be made available on request.
